# Supplementary material for: Context-aware deep learning enables high-efficacy localization of high concentration microbubbles for super-resolution ultrasound localization microscopy
Source: Nat Commun. 2024 Apr 4;15:2932. doi: 10.1038/s41467-024-47154-2 (PMC10995206; doi:10.1038/s41467-024-47154-2)
Supplement: Supplementary file 1 — Supplementary Information [file 41467_2024_47154_MOESM1_ESM.pdf]

Supplementary Methods

1. Field II phase-aberrated microbubble (MB) simulation

To study the effect of propagation of localization error from conventional localization, we simulated 400 images of phase-aberrated MBs using Field II. The Field II simulation sequence was configured according to the CAM imaging settings described in Supplementary Table 1. In each image, single MBs were randomly positioned within a 2D field that was 10 mm wide, extending from z = 2 mm to z = 15 mm in depth, and with a pixel size of 0.064 λ. We simulated phase aberrations in individual MBs by introducing perturbations to the phase/speed of sound in the RF signal. Random phase delays for each element were sampled from a uniform distribution between 0 and π radians. The corresponding time delay based on the central frequency (20 MHz) was then applied to the transmit and receive delay for each element. We used conventional localization to extract Field II MB templates for training the LOCA-ULM network. The localization accuracy of both the conventional method and LOCA-ULM was compared against the ground truth positions (Supplementary Fig. 1). Localization errors exceeding 98.56 μm was excluded from the analysis.

2. Gaussian and Field II MB Modeling

To study the effect of MB modeling on the training data, we created two additional MB templates: Gaussian and Field II. For the Gaussian MB simulation, 500 MB signals were extracted from the *in vivo* data and was fitted with a two-dimensional Gaussian model to determine the standard deviations along the lateral and axial axes (σ_x and σ_z). The parameter range for the Gaussian MB simulation was computed based on the mean and standard deviations of σ_x and σ_z. We set boundaries at two standard deviations from the mean for both σ_x and σ_z to capture the central 95% of the distribution while excluding outliers. When generating MB signals in simulation, the 2D Gaussian distribution was established by randomly drawing σ_x and σ_z from an uniform distribution ($\sigma_x \in [43 - 76\ \mu m]; \sigma_z \in [26 - 60\ \mu m]$) for CAM imaging and ($\sigma_x \in [66 - 127\ \mu m]; \sigma_z \in [43 - 74\ \mu m]$) for rat brain imaging. For the Field II simulation, we distributed 30 microbubbles randomly in a two-dimensional domain that is 5 mm wide and extends from z = 2 mm to z = 12 mm in depth. The simulation data were interpolated to a resolution of 0.064 λ pixel size for the CAM images and 0.1 λ pixel size for rat brain images (Supplementary Table I, MB template pixel resolution) and repeated 100 times to extract 3000 Field II MB templates. The Field II simulation sequence was configured according to the CAM and rat brain ultrasound imaging settings described in Supplementary Table 1.

3. Deep-ULM and mSPCN implementations

This study employed two additional deep learning-based ULM models for comparative analysis against LOCA-ULM: Deep-ULM and mSPCN. Deep-ULM used the U-Net architecture where the encoder consists of three stages, each containing two convolutional layers with 3×3 kernels, batch normalization, leaky ReLU activation, and a 2×2 MaxPooling operation. The latent layer, positioned between the encoder and the decoder, includes two convolutional layers with 3×3 kernels and a dropout layer (probability 50%). The decoder consists of three stages with deconvolution layers with 5×5 kernels (first layer with a stride of 2 and the second a stride of 1), batch normalization, and leaky ReLU activation. The first two stages of the decoder also incorporate a 2×2 upsampling layer. The final layer is a single-channel output convolutional layer with linear activation. The mSPCN model (provided by the authors in DOI: 10.21227/jdgd-0379) employs a sub-pixel architecture that consists of 13 convolution layers. It begins with a 9×9 kernel convolution layer for feature extraction, followed by ten 3×3 convolution kernel convolution layers. Residual blocks are integrated every two layers. The 12th convolution layer forms a global residual

connection to the first layer, while the final layer performs the upscaling operation using a sub-pixel convolution layer. We trained both networks using the Adam optimizer with a learning rate of 0.001 for 400 epochs, aiming to minimize the following cost function:

$$\mathcal{L}(x, y|\theta) = \|f(x|\theta) - \lambda_0 y\|_2^2 + \lambda_1 \|f(x|\theta)\|_1$$

where x denotes the simulated ultrasound MB image, and y represents the super-resolved image. The coefficients were set as $\lambda_0 = 100$ and $\lambda_1 = 0.1$ for both networks. The mSPCN and Deep-ULM models operate as image-to-image translation networks, where the output depicts the center of MB as a cluster of activated pixels rather than a single pixel. In line with the method outlined in mSPCN, the MB positions were determined by applying threshold to remove constant background and a 2D Gaussian kernel to group the adjacent non-zero pixels. The centroid is identified by computing the center of mass. The training datasets for LOCA-ULM, Deep-ULM, and mSPCN were generated using the same MB flow simulation (brightness, lifetime, and velocity) and MB templates (i.e., LSGAN, Gaussian, and Field II MB templates).

4. Assessment of depth-dependent MB signal variations for LOCA-ULM training

To investigate the field-of-view (FOV) dependent spatial variations in MB signals, we employed materials that mimic the acoustic attenuation of real tissues. This approach enabled us to replicate the *in vivo* conditions where microbubbles exhibit variations due to frequency-dependent attenuation. We used a 60:40 volume ratio mixture of condensed milk and water to create a solution that both has a similar acoustic attenuation to brain tissue, specifically 0.50 dB/cm/MHz[1,2], and is a liquid environment to allow even distributions of MBs at different imaging depths.

The attenuation coefficient was measured using a simple experimental setup as illustrated in Supplementary Fig. 8a below, where two identical CTS 10MHz IS1004HR transducers were positioned 5.5 cm apart to operate in a “pitch-and-catch” mode to assess ultrasound signal attenuation through propagation in the sample material contained in the testing cell. An ultrasound pulser (Olympus 5800 PR) was used to drive the transmitting transducer and a DAQ (NI PXI5124) was used to sample the ultrasound signal acquired at the receiving transducer. The attenuation coefficient α was calculated by:

$$\alpha = \frac{1}{f}\frac{20}{d}\log_{10}\left(\frac{A_0}{A}\right)$$

where d is the propagation distance (i.e., 5.5 cm), f is the ultrasound frequency (10 MHz), and A_0, A are the amplitude corresponding to the peak of the received signal for pure water and condensed milk-water mixture, respectively. The experiment was conducted twice, yielding an attenuation coefficient of approximately 0.50 dB/cm/MHz and 0.51 dB/cm/MHz, which is similar to the attenuation coefficient of brain tissue[1,2].

After validating the attenuation coefficient, activated Definity MBs were diluted by a factor of 5000 in distilled water. 10 μL of this diluted solution was carefully mixed with degassed condensed milk-water mixture, resulting in a final volume of 100 mL. We then acquired experimental MB data using the same imaging setup as the *in vivo* rat brain imaging study. To analyze the MB signals, we divided the FOV into three regions, corresponding to three depth segments: 2-5 mm, 5-8 mm, and 8-11 mm (Supplementary Fig. 8b). A total of 3780 individual MB signals were extracted across all imaging zones followed by full width at half maximum (FWHM) measurements respect to the identified center (in both the axial and lateral dimensions) for the analysis of MB signal distribution.

In Supplementary Fig. 8c, the axial FWHM of experimental MBs varies from 134.97 μm to 149.66 μm, while the lateral FWHM varies from 192.12 μm to 247.87 μm from the shallowest to the deepest regions. We subsequently trained the LSGAN model using 3780 experimental MB signals and generated an equal number of synthetic MB signals. As shown in Supplementary Fig. 8d, the distribution of LSGAN-generated MBs closely follows the distribution of the experimental MBs, as evidenced by the similar average lateral and axial FWHM values for LSGAN-generated MBs ($\mu_x = 215.48\, \mu m$, $\mu_z = 144.87\, \mu m$) and experimental MBs ($\mu_x = 218.69\, \mu m, \mu_z = 144.34\, \mu m$). Through this experiment, we show that the LSGAN-generated MB templates encompass the MB signal distribution across the entire ultrasound FOV, thereby enhancing the robustness of LOCA-ULM.

Supplementary Figures

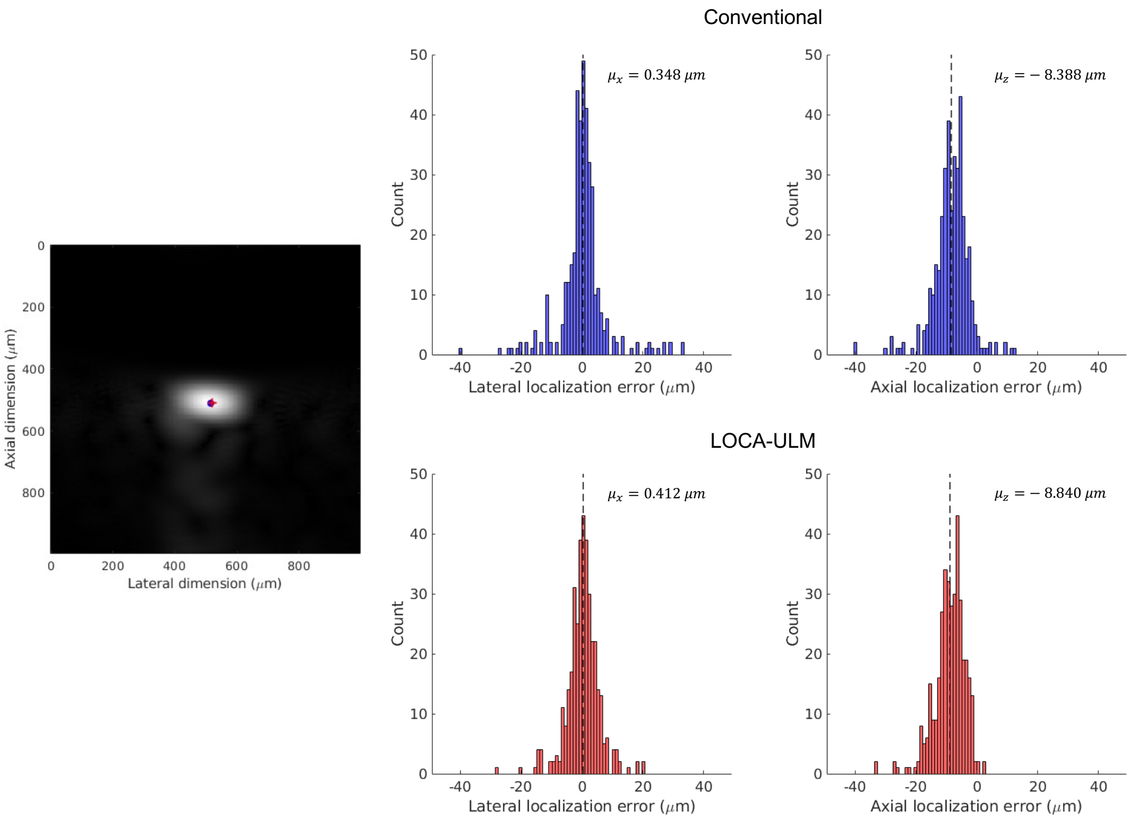

Supplementary Fig 1. Comparative analysis of LOCA-ULM and conventional localization methods using a Field II simulated test set with induced phase aberration. The histograms show the distribution of the lateral and axial localization errors respect to the ground truth positions (n = 400 phase-aberrated MB images). Source data are provided as a Source Data file.

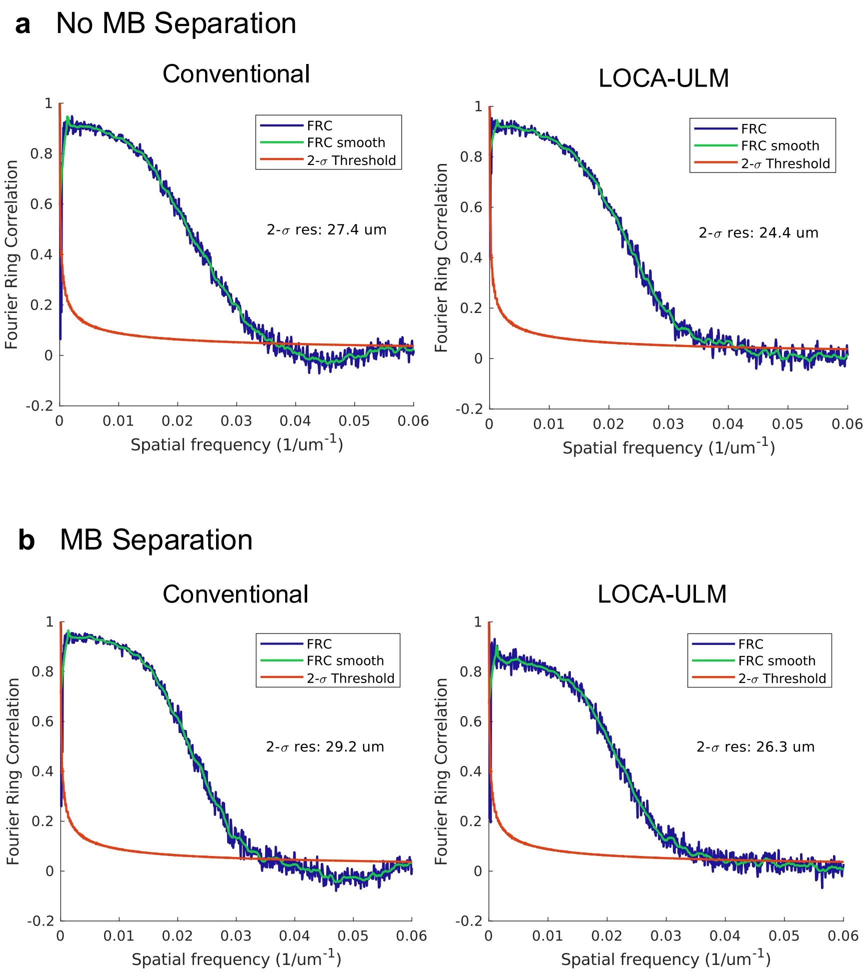

Supplementary Fig 2. FRC curve using 2-σ threshold for four ULM reconstruction. **a** Conventional and LOCA-ULM without MB separation. **b** Conventional and LOCA-ULM using MB separation. Source data are provided as a Source Data file.

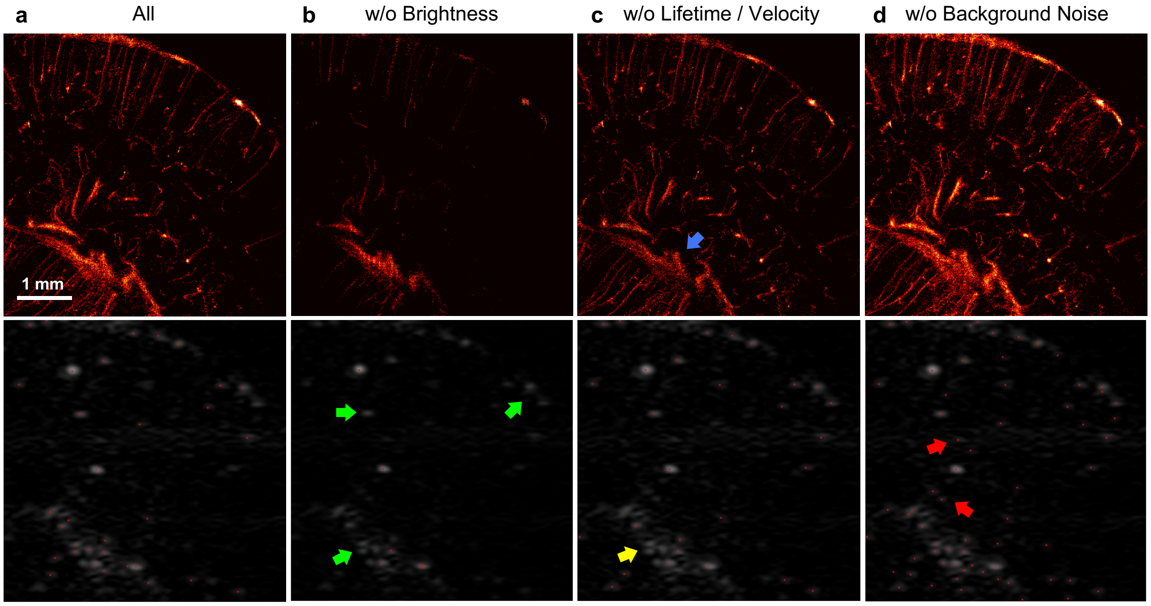

Supplementary Fig 3. Ablation study of MB signal characteristics in the LOCA-ULM simulation framework. Top row: **a** ULM images with all MB characteristics included (All), **b** without brightness variations (w/o Brightness), **c** without MB movement and lifetime (w/o Lifetime/Velocity), and **d** without simulated background ultrasound noise (w/o Background Noise). Bottom row: *in vivo* rat brain contrast-enhanced B-mode image and localization results under each ablation scenario.

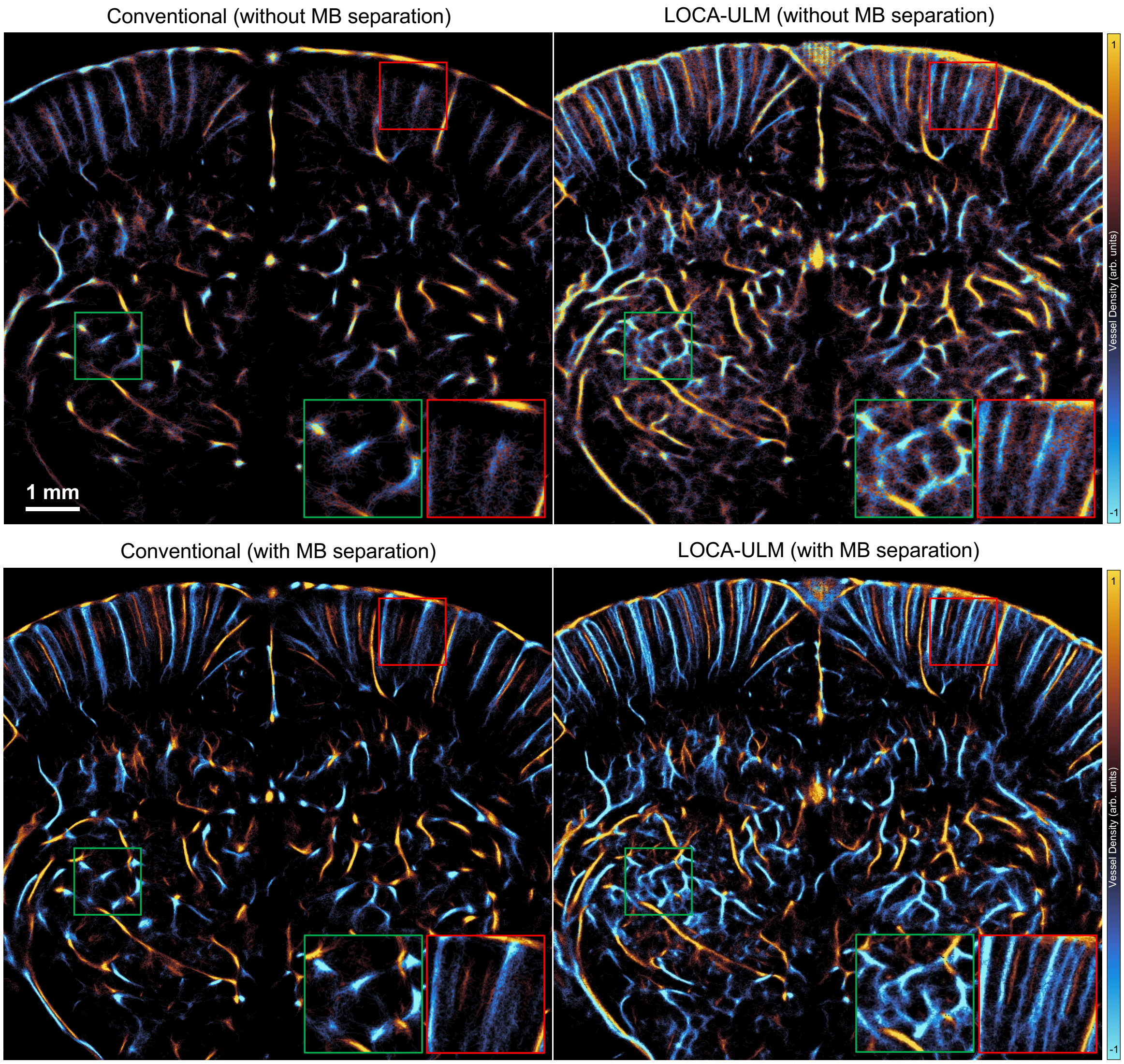

Supplementary Fig 4. Comparison of conventional ULM and LOCA-ULM with MB separation at high MB concentration. Each ULM images were generated by accumulating 16000 frames of ultrasound data (a total of 20 seconds of acquisition) for MB injection rate of 90 $\mu L/\text{min}$

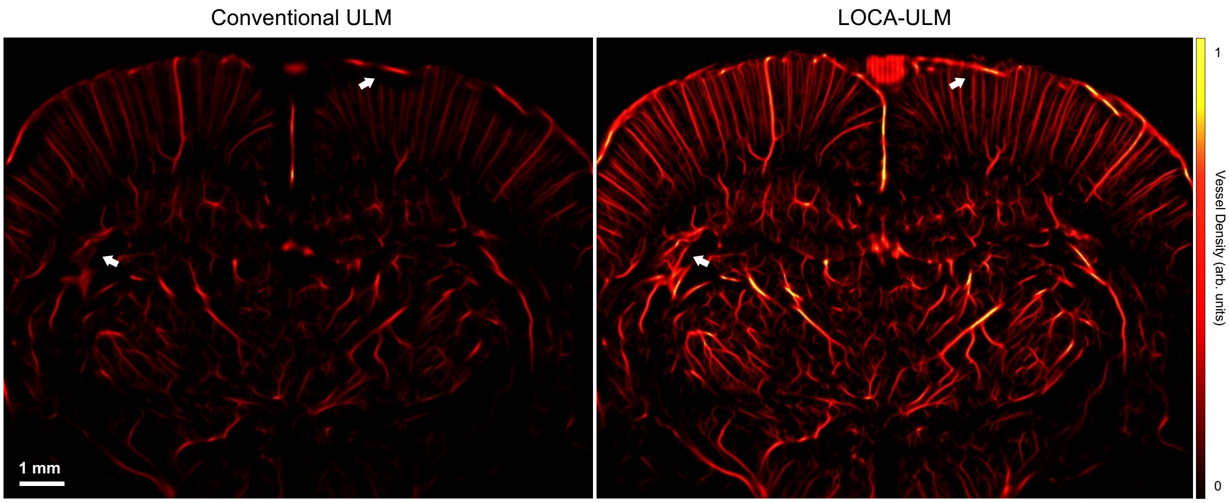

Supplementary Fig 5. Comparison of conventional ULM and LOCA-ULM for functional ULM (fULM) experiment. Each ULM images were generated by accumulating 720000 frames of ultrasound data for MB injection rate of 60 $\mu L/\mathrm{min}$.

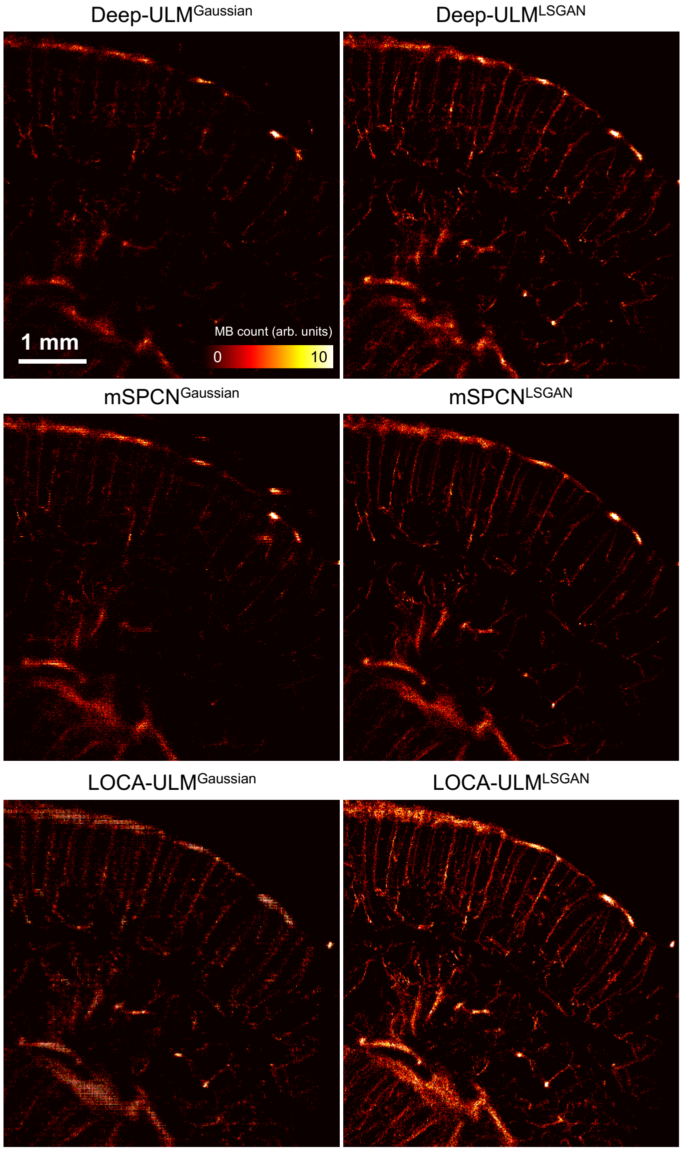

Supplementary Fig 6. Comparison of Deep-ULM, mSPCN, and LOCA-ULM trained with simulation data generated using either Gaussian- or LSGAN-based MB templates. *In vivo* rat brain imaging data were used for testing.

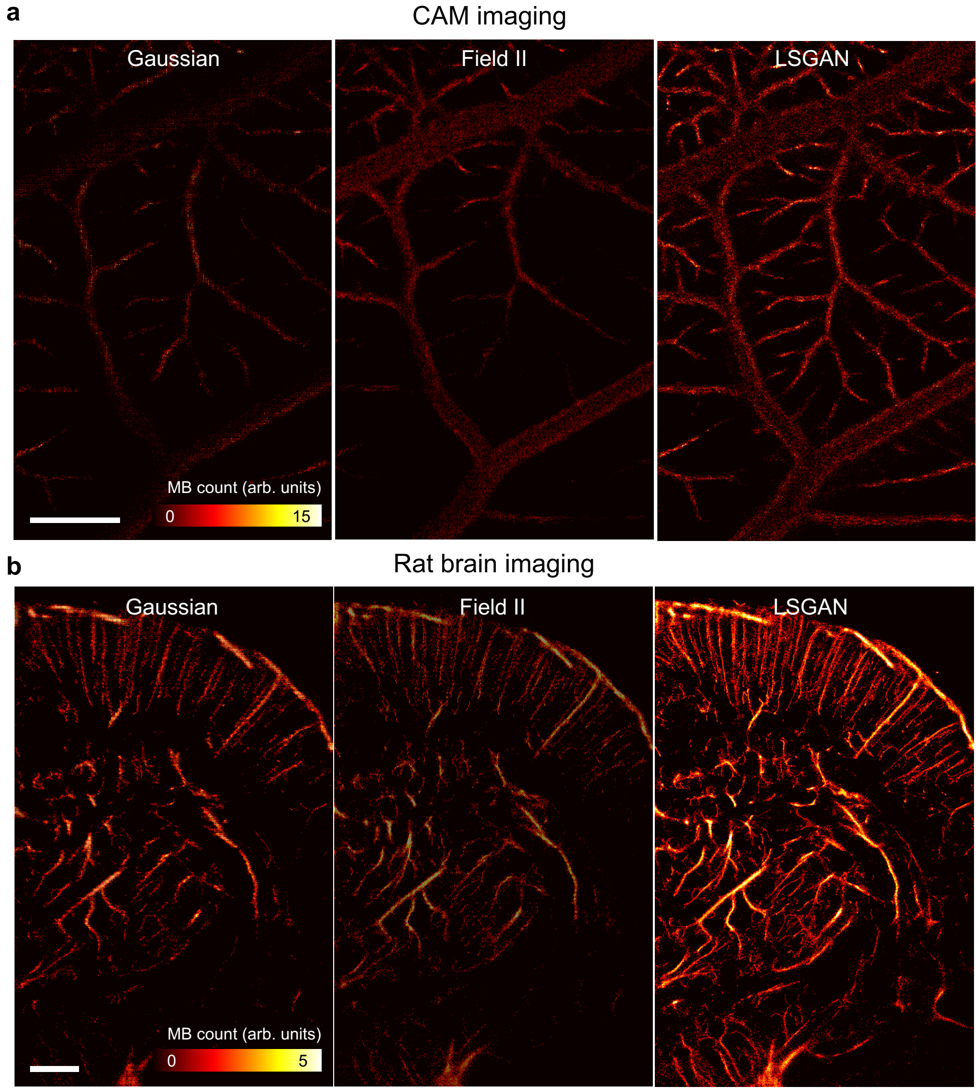

Supplementary Fig 7. Comparison of reconstructed ULM images using different MB simulation methods: Gaussian, Field II, and LSGAN. a ULM imaging results in the CAM study, **b** ULM imaging results in the rat brain study. The ULM images demonstrate the impact of MB model selection on image quality, contrast, and artifact presence in ULM. Scalebar represents 1 mm.

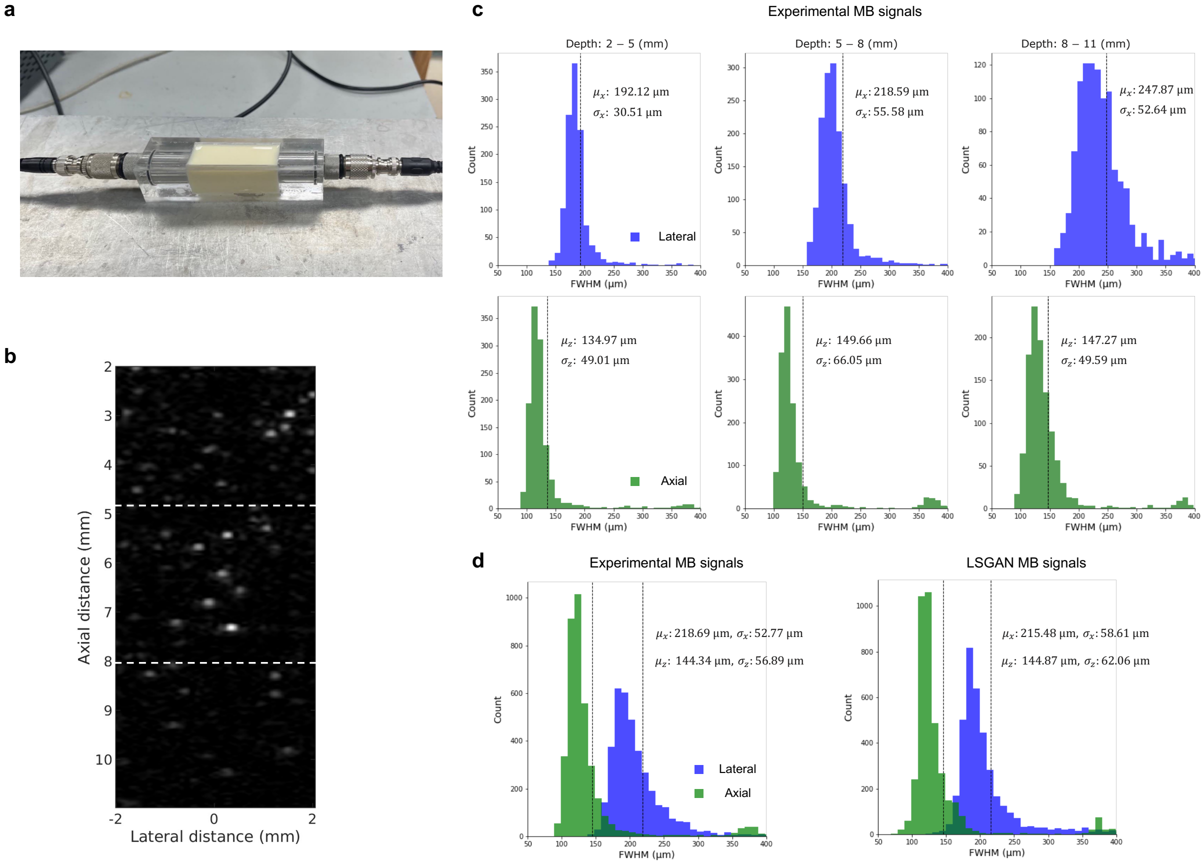

Supplementary Fig 8. Experiment setup for attenuation coefficient measurement and experimental MB signal analysis a A custom-built housing with a testing cell sandwiched between two CTS 10MHz IS1004HR transducers operating in a "pitch-and-catch" mode. A mixture of condensed milk and water was used as the testing medium to simulate rat brain tissue. **b** B-mode ultrasound image displaying MBs suspended within the testing medium. White dashed lines denote the three analyzed depth zones. **c** Histograms showing the full width at half maximum (FWHM) of MB signals in lateral (blue) and axial (green) dimensions across different depths. **d** Histograms showing the FWHM for experimental MB signals across entire FOV (n = 3780 localized MBs) and LSGAN-generated MB signals (n = 3780 synthetic MBs). Source data are provided as a Source Data file.

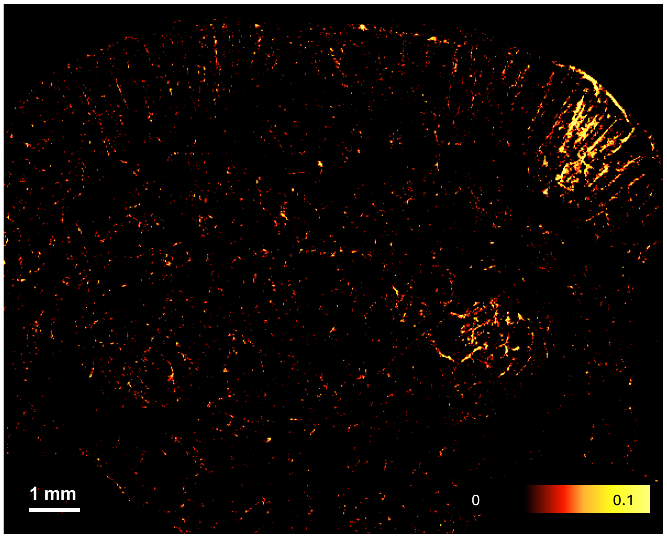

Supplementary Fig 9. Regions with positive correlation identified by LOCA-ULM during functional ULM (fULM) imaging. The map highlights areas with increased blood flow correlating to whisker stimulation, with a color scale representing the Pearson's correlation coefficient.

Supplementary Tables

Table 1

In vivo study acquisition parameters, microbubble concentrations, and imaging resolution

	**CAM**	**Rat Brain**		
	**Standard Imaging**	**Standard Imaging**	**fULM Imaging**	**fUS Imaging**
***In vivo* acquisition parameters**				
Transducer type	L35-16vX	L22-14vX	L22-14vX	L22-14vX
Center Frequency	20 MHz	15.625 MHz	15.625 MHz	15.625 MHz
Sampling Frequency	125 MHz	62.5 MHz	62.5 MHz	62.5 MHz
Wavelength	77 μm	98.56 μm	98.56 μm	98.56 μm
No. of compounding angles	9	5	5	9
Step size	1°	1°	2°	1°
Voltage	6V	6V	6V	40V
**Concentration**				
	70 μL Bolus injection	15, 20, 30, 40, 90 $\mu L/min$	60 $\mu L/min$	Contrast-free
**Image resolution**				
MB template pixel resolution	4.928 μm	9.856 μm	9.856 μm	
DECODE network input pixel resolution	9.856 μm	19.712 μm	19.712 μm	
DECODE network output pixel resolution	4.928 μm	9.856 μm	9.856 μm	

Supplementary References

1. Aubry, J.-F. et al. ITRUSST Consensus on Biophysical Safety for Transcranial Ultrasonic Stimulation. *arXiv preprint arXiv:2311.05359* (2023).
2. Duck, F. Physical properties of tissues: a comprehensive reference book. (Academic press, 2013).
