# Supplementary material for: Context-aware deep learning enables high-efficacy localization of high concentration microbubbles for super-resolution ultrasound localization microscopy
Source: Nat Commun. 2024 Apr 4;15:2932. doi: 10.1038/s41467-024-47154-2 (PMC10995206; doi:10.1038/s41467-024-47154-2)
Supplement: Supplementary file 3 — Reporting Summary - NEW [file 41467_2024_47154_MOESM3_ESM.pdf]

nature portfolio

Corresponding author(s): Pengfei Song

Last updated by author(s): Mar 4, 2023

Reporting Summary

Nature Portfolio wishes to improve the reproducibility of the work that we publish. This form provides structure for consistency and transparency in reporting. For further information on Nature Portfolio policies, see our Editorial Policies and the Editorial Policy Checklist.

Statistics

For all statistical analyses, confirm that the following items are present in the figure legend, table legend, main text, or Methods section.

n/a	Confirmed	
☐	☒	The exact sample size (n) for each experimental group/condition, given as a discrete number and unit of measurement
☐	☒	A statement on whether measurements were taken from distinct samples or whether the same sample was measured repeatedly
☒	☐	The statistical test(s) used AND whether they are one- or two-sided<br>*Only common tests should be described solely by name; describe more complex techniques in the Methods section.*
☒	☐	A description of all covariates tested
☒	☐	A description of any assumptions or corrections, such as tests of normality and adjustment for multiple comparisons
☐	☒	A full description of the statistical parameters including central tendency (e.g. means) or other basic estimates (e.g. regression coefficient) AND variation (e.g. standard deviation) or associated estimates of uncertainty (e.g. confidence intervals)
☒	☐	For null hypothesis testing, the test statistic (e.g. F, t, r) with confidence intervals, effect sizes, degrees of freedom and P value noted<br>*Give P values as exact values whenever suitable.*
☒	☐	For Bayesian analysis, information on the choice of priors and Markov chain Monte Carlo settings
☒	☐	For hierarchical and complex designs, identification of the appropriate level for tests and full reporting of outcomes
☐	☒	Estimates of effect sizes (e.g. Cohen's d, Pearson's r), indicating how they were calculated

Our web collection on statistics for biologists contains articles on many of the points above.

Software and code

Policy information about availability of computer code

Data collection	Ultrasound raw RF data were acquired using a Vantage ultrasound system 4.7.6 (Verasonics, Kirkland, WA, USA) and custom Matlab R2021b (Mathworks, Cambridge, MA, USA) codes were used to collect the data. Optical images of the chorioallantoic membrane (CAM) were obtained using a Nikon SMZ800 stereomicroscope (Nikon, Tokyo, Japan) with A DS-Fi3 digital camera (5.9-Mpixel CMOS image sensor, Nikon).
Data analysis	Python 3.8.13 was used to construct LOCA-ULM, Deep-ULM, and mSCPN models. The code to generate LSGAN-based microbubble signals and microbubble simulation pipeline is available at (https://github.com/illyrs2/LOCA-ULM). The previously published DECODE software (available on github.com/TuragaLab/DECODE) was used in this study. ULM data processing and analysis were conducted using custom codes built with Matlab R2022a (MathWorks, Cambridge, MA, USA). GIMP was used to obtain segmentation of CAM optical images. Activation maps were overlayed (using 'opacity') to structural ULM images using Adobe Photoshop 2023.

For manuscripts utilizing custom algorithms or software that are central to the research but not yet described in published literature, software must be made available to editors and reviewers. We strongly encourage code deposition in a community repository (e.g. GitHub). See the Nature Portfolio guidelines for submitting code & software for further information.

Data

Policy information about availability of data

All manuscripts must include a data availability statement. This statement should provide the following information, where applicable:

- Accession codes, unique identifiers, or web links for publicly available datasets
- A description of any restrictions on data availability
- For clinical datasets or third party data, please ensure that the statement adheres to our policy

The data for Figures 3, 4, and 5 used in this study are available at Zenodo at [DOI: 10.5281/zenodo.10711806]. Data for Figure 6 is available from the corresponding author upon request. Source data are provided with this paper.

Research involving human participants, their data, or biological material

Policy information about studies with human participants or human data. See also policy information about sex, gender (identity/presentation), and sexual orientation and race, ethnicity and racism.

Reporting on sex and gender	This study does not involve human participants or human data.
Reporting on race, ethnicity, or other socially relevant groupings	This study does not involve human participants or human data.
Population characteristics	This study does not involve human participants or human data.
Recruitment	This study does not involve human participants or human data.
Ethics oversight	This study does not involve human participants or human data.

Note that full information on the approval of the study protocol must also be provided in the manuscript.

Field-specific reporting

Please select the one below that is the best fit for your research. If you are not sure, read the appropriate sections before making your selection.

☒ Life sciences ☐ Behavioural & social sciences ☐ Ecological, evolutionary & environmental sciences

For a reference copy of the document with all sections, see nature.com/documents/nr-reporting-summary-flat.pdf

Life sciences study design

All studies must disclose on these points even when the disclosure is negative.

Sample size	This study was designed to compare microbubble localization performance across different algorithms. The sample size was determined to include a range of microbubble concentrations across both simulation and in vivo studies. This analysis includes hundreds of thousands of microbubble events in simulation and tens of millions in in vivo. To train the network, the simulated frames was selected to be sufficiently large, ensuring minimization of statistical errors.
Data exclusions	Pilot experiments (N=2) were conducted for protocol optimization purposes, beyond which no data were excluded.
Replication	For in sillico studies, we used the LOCA-ULM simulation pipeline to generate synthetic datasets, which is accurately replicated using consistent parameters. For the in vivo chicken embryo chorioallantoic membrane and rat brain imaging studies, specific experimental details are provided in the Methods section. We have replicated rat brain experiments across different animals using different microbubble concentrations. Moreover, our study is designed to compare localization performance of different algorithms using the same dataset, rather than comparing between repeated imaging studies.
Randomization	Samples were not subjected to randomization because this study focuses on presenting an image analysis pipeline.
Blinding	Data analysis was conducted and evaluated by computer algorithms, independent of human intervention. Blinding in data analysis was not necessary, as it involved no grouping.

Reporting for specific materials, systems and methods

We require information from authors about some types of materials, experimental systems and methods used in many studies. Here, indicate whether each material, system or method listed is relevant to your study. If you are not sure if a list item applies to your research, read the appropriate section before selecting a response.

Materials & experimental systems

n/a	Involved in the study
☒	☐ Antibodies
☒	☐ Eukaryotic cell lines
☒	☐ Palaeontology and archaeology
☐	☒ Animals and other organisms
☒	☐ Clinical data
☒	☐ Dual use research of concern
☒	☐ Plants

Methods

n/a	Involved in the study
☒	☐ ChIP-seq
☒	☐ Flow cytometry
☒	☐ MRI-based neuroimaging

Animals and other research organisms

Policy information about studies involving animals; ARRIVE guidelines recommended for reporting animal research, and Sex and Gender in Research

Laboratory animals	Four female Sprague-Dawley rats were used for this study (8, 9, 10, 12 weeks-old). The animals were obtained from the Charles River Laboratories, Inc. Fertilized chicken eggs (white leghorn) were obtained from the University of Illinois Poultry Research Farm for chorioallantoic membrane (CAM) imaging.
Wild animals	This study did not involve wild animals.
Reporting on sex	Only female animal were used in this study.
Field-collected samples	This study did not involve samples collected from the field.
Ethics oversight	All experimental procedures were conducted in accordance with the guidelines set by the University of Illinois Urbana-Champaign, Institutional Animal Care and Use Committee (IACUC Protocol number #22165).

Note that full information on the approval of the study protocol must also be provided in the manuscript.

Plants

Seed stocks	*Report on the source of all seed stocks or other plant material used. If applicable, state the seed stock centre and catalogue number. If plant specimens were collected from the field, describe the collection location, date and sampling procedures.*
Novel plant genotypes	*Describe the methods by which all novel plant genotypes were produced. This includes those generated by transgenic approaches, gene editing, chemical/radiation-based mutagenesis and hybridization. For transgenic lines, describe the transformation method, the number of independent lines analyzed and the generation upon which experiments were performed. For gene-edited lines, describe the editor used, the endogenous sequence targeted for editing, the targeting guide RNA sequence (if applicable) and how the editor was applied.*
Authentication	*Describe any authentication procedures for each seed stock used or novel genotype generated. Describe any experiments used to assess the effect of a mutation and, where applicable, how potential secondary effects (e.g. second site T-DNA insertions, mosiacism, off-target gene editing) were examined.*
